# Transcription Factors of the GLRs Family Are Involved in Cytokinin-Dependent Regulation of Plastid RNA Polymerase *SCA3* Gene Expression during Deetiolation of *Arabidopsis thaliana*

**DOI:** 10.1134/S1607672922050040

**Published:** 2022-10-27

**Authors:** A. S. Doroshenko, A. M. Malyukova, M. N. Danilova, Vl. V. Kuznetsov, V. V. Kusnetsov

**Affiliations:** 1 Timiryazev Institute of Plant Physiology, Russian Academy of Sciences, Moscow, Russia; 2 Moscow State University, Moscow, Russia

**Keywords:** *Arabidopsis thaliana*, deetiolation, cytokinins, transcription factors, gene expression, chloroplast biogenesis

## Abstract

Light-dependent transcription factors GLKs of *Arabidopsis thaliana* are involved in the anterograde regulation of chloroplast biogenesis during deetiolation: they regulate the expression of photosynthetic nuclear-encoded genes and also mediate the transcription of plastid genes. Chloroplast biogenesis is determined at the same time by light and by endogenous factors (phytohormones), among which cytokinins significantly accelerate the formation of photosynthetically active chloroplasts. In this work, it was shown that *trans*-factors GLKs function as cytokinin-dependent regulators, mediating the positive cytokinin effect on the plastome expression through the activation of transcription of the *SCA3* nuclear gene encoding the plastid RNA polymerase RPOTp.

## INTRODUCTION

Despite the key role of chloroplasts in plant life, the molecular mechanisms of their biogenesis remain poorly understood. Chloroplasts are usually formed either from proplastids in meristematic tissues or from etioplasts during plant deetiolation.

The main exogenous factor that determines the biogenesis of photosynthetically active chloroplasts from etioplasts is light [1]. Light of different quality is perceived by one or another group of receptors (primarily in the cytoplasm). Then, the signal enters the nucleus, which leads to a large-scale change in the expression of plant nuclear genes, many of which encode structural and regulatory proteins of chloroplasts. The control of the biogenesis of photosynthetically active plastids by nuclear coding proteins is called anterograde regulation, which probably plays the key role at all stages of chloroplast biogenesis.

One of the most striking examples of anterograde control of chloroplast formation is the reprogramming of plastid gene expression as a result of light- and hormone-dependent changes in the activity of the plastid transcription apparatus during deetiolation. In etioplasts, transcription is performed mostly by RNA polymerases of the NEP type (Nuclear-Encoded RNA Polymerase)—RPOTp and, to a lesser extent, RPOTmp, which largely transcribe housekeeping genes. The second multisubunit RNA polymerase PEP (Plastid-Encoded RNA Polymerase) consists of core subunits α, β, β', and β" and exhibits weak transcriptional activity in non-photosynthetic plastids. During deetiolation, the PEP polymerase undergoes significant structural changes due to the formation of a complex with one of the sigma factors (SIG1-SIG6) and nuclear coding proteins associated with PEP (PAP1-PAP12), after which the PEP polymerase initiates transcription of photosynthetic plastome genes [2]. Thus, the nuclear genome controls the transcriptional activity of the plastid genome by coordinating the transcription of the genes encoding RNA polymerases RPOTp and RPOTmp, sigma factors, and PAP proteins.

The key regulators of nuclear genome expression are the light-dependent transcription factors. In *A. thaliana*, two *trans*-factors—GLK1 and GLK2 (Golden two-LiKe)—were identified, the inactivation of which leads to disturbances in chloroplast biogenesis [3] due to a decrease in the transcription of nuclear genes encoding chlorophyll biosynthesis enzymes and proteins of photosynthetic complexes and thylakoid membranes [4].

In addition to light, the deetiolation program is determined by endogenous factors—phytohormones, among which cytokinins (CKs) can accelerate the formation of chloroplasts [5]. It was convincingly shown that CKs upregulate the expression of genes encoding the chlorophyll biosynthesis enzymes and proteins of the plastome transcription machinery, stimulate the accumulation of photosynthetic pigments, and accelerate the formation of chloroplast ultrastructure [6, 7]. In a number of tests, a synergistic effect in the action of light and CKs is observed, which allows for the possibility of intersection of light and cytokinin signal transduction pathways [6–8]. To date, it has been shown that one of the “crossing points” is the light-dependent *trans*-factor HY5, the inactivation of which leads to a decrease in the positive effect of CKs during deetiolation [8]. However, the absence of HY5 does not abolish the effect of CKs, which suggests the involvement of other light-dependent *trans-*factors that mediate the effect of CKs during deetiolation [9].

As already mentioned, in *A. thaliana*, the inactivation of the genes encoding two *trans*-factors GLK1 and GLK2 leads to disruption of transcription of both nuclear and plastid photosynthetic genes [3, 4]. In addition, Kobayashi et al. [10] showed that proteins GLKs are involved in the realization of the positive effect of CKs on the accumulation of chlorophyll during root deetiolation, and the *GLK2* gene expression is activated by the hormone. These results suggest that GLKs may be involved in the regulation of the expression of nuclear genes of the plastid transcription apparatus by light and CKs. In this work, we demonstrated for the first time the involvement of *trans*-factors GLKs in the cytokinin-dependent regulation of the expression of the nuclear *SCA3* gene encoding plastid RNA polymerase, which leads to changes in the expression of plastid genes.

## MATERIALS AND METHODS

The objects of the study were wild-type *Arabidiopsis thaliana* plants of the Columbia-0 ecotype and its knockout mutant for the *trans*-factor genes *glk1glk2* (N9807, NASC, United Kingdom). The presence of the dSpm insertion in the *GLK1* and *GLK2* genes [3] was confirmed by PCR using primers flanking the insert: for the *GKL1* gene: *Spm5* (F) 5'-ggatccgacactctttaattaactgacact-3'; (R) 5'-acttcttcaccttttccccgaacta-3'; for the *GLK2* gene: *Spm1* (F) 5'-cctatttcagtaagagtgtggggttttgg-3'; (R) 5'-aacaatctttacttttcttccctttacg-3'. PCR analysis of the *glk1glk2* knockout mutant DNA confirmed the presence of dSpm inserts in the *GLK1* and *GLK2* genes. Amplification of DNA from the wild-type plants showed the absence of dSpm constructs in the maternal *A. thaliana* line. Thus, the homozygosity of *A. thaliana glk1glk2* knockout lines obtained from the NASC seed bank was confirmed.

To study the involvement of the *trans*-factors GLK1 (*AT2G20570*) and/or GLK2 (*AT5G44190*) in the cytokinin-dependent regulation of the *SCA3* gene expression during deetiolation, we used the experimental protocol developed by Chory et al. [11]. Seeds of wild-type *A. thaliana* and *glk1glk2* knockout mutant were sterilized with sodium hypochlorite solution and seeded on Petri dishes with Murashige–Skoog nutrient medium containing 1/2 nutrients (Duchefa, Netherlands) without sucrose and cytokinin or with the addition of *trans*-zeatin (1 µM). The seeds were stratified for 4 days at 4°C, after which the Petri dishes were incubated at 22°C in complete darkness. Four days after the moment of germination, the plants were fixed in liquid nitrogen under weak green light (5 ± 2 µmol s^–1^ m^–2^). The rest of the seedlings were transferred to an MLR-352Н-PE climatic chamber (Sanyo, Japan) illuminated with white light with an intensity of 120 µmol s^–1^ m^–2^ and fixed in liquid nitrogen after 6 and 16 h of incubation.

The relative level of transcripts was assessed by real-time PCR after reverse transcription (RT-PCR) using a LigthCyclerR96 thermocycler (Roche, Switzerland). The number of target gene transcripts was normalized relative to the mRNA content of the reference polyubiquitin gene *UBQ10* (*AT4G05320*). The following primer pairs were used for real-time PCR analysis: *ARR5*: (F) ctactcgcagctaaaacgc, (R) gccgaaagaatcaggaca; *GLK1*: (F) tcggactaaaaatggatggcttg, (R) ggtagaaggcggaggtaagtgtttg; *GLK2*: (F) gccaaaacacaagcctaatactccg, (R) tgtggatagagtggttgctgatgc; *SCA3*: (F) ttgctgctgcttgctattctgc, (R) gcacaatcaccaagccaact; *accD*; (F) gctaccaatcaatgtttacctc, (R) gattgataatcacataaaaccg; *clpP*: (F) cattccagatattacccatcca, (R) gccaagaggttgataccgaa; and *UBQ10*: (F) gcgtcttcgtggtggttctaa, (R) gaaagagataacaggaacggaaaca. The samples were analyzed in three biological replicates, each of which included three or four analytical replicates. Data were statistically processed using Student’s *t* test (*p < 0.05, **p < 0.01).

## RESULTS AND DISCUSSION

**Fig. 1.**
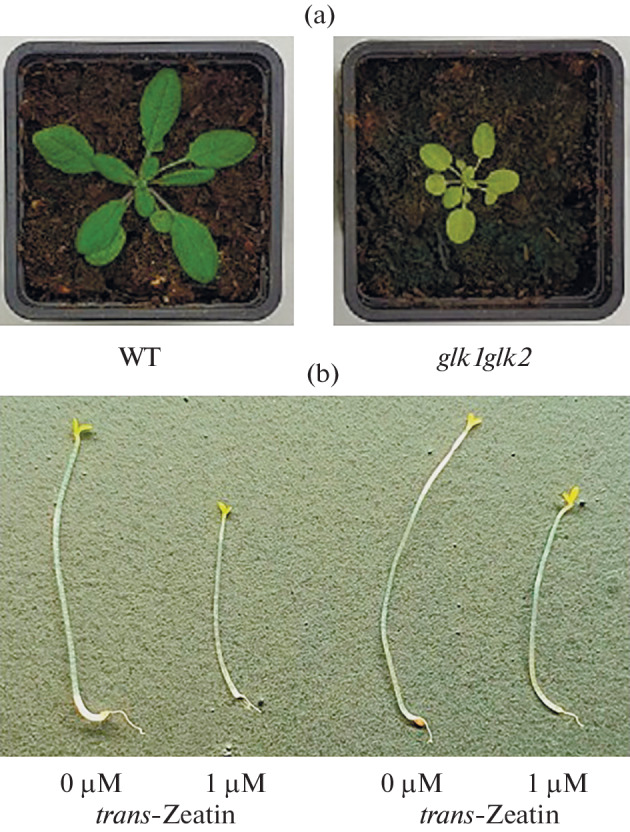
Morphology of *A. thaliana* wild-type and *glk1glk2* knockout mutant plants. (a) Three-week-old plants, wild type (WT) on the left, *glk1glk2* knockout-mutant on the right; (b) 4-day-old etiolated wild-type and *glk1glk2* seedlings grown on nutrient medium without hormone (0 μM *trans*-zeatin) or in the presence of CK (1 μM *trans*-zeatin) after 16 h of illumination.

**Effect of cytokinin on the morphology of the wild-type seedlings and the** ***glk1glk2*** **knockout mutant of** ***A. thaliana*.** The GLKs family of proteins includes two transcription factors, GLK1 and GLK2, whose functions considerably overlap. The first-order knockout mutants have weak phenotypic differences from the wild-type plants, whereas the double mutant *glk1glk2*, which we used in this study, differs from the maternal line in a smaller rosette size and a reduced chlorophyll content (Fig. 1a) [3].

One of the characteristic effects of CKs is the shortening of the seedling hypocotyl in the dark [11]. Under our experimental conditions, seedlings of the wild type and the *glk1glk2* double knockout mutant grown on a nutrient medium without CKs did not differ phenotypically (Fig. 1b). The addition of *trans*-zeatin (1 μM) to the growth medium resulted in the suppression of hypocotyl growth in the wild-type plants by 54% (in the absence of the CK, 12.6 ± 1.5 mm; in the presence of *trans*-zeatin, 6.88 ± 1.6 mm). Plants of the *glk1glk2* knockout mutant in the presence of CK had a hypocotyl length 62% shorter than the seedlings grown without the hormone (in the absence of the CK, 12.9 ± 1.4 mm; in the presence of *trans*-zeatin, 8.11 ± 1.45 mm). This result confirms the effectiveness of the treatment with the CK under the our experimental conditions and indicates the sensitivity of the mutant to the exogenous hormone.

**Mutations for** ***trans*-factor genes** ***GLKs*** **(*glk1glk2*) do not disturb the sensitivity of plants to cytokinin**. To confirm the sensitivity of the *glk1glk2* knockout mutant to the exogenous CK, we studied the dynamics of the content of transcripts of the *ARR5* gene belonging to the family of cytokinin-A response regulators. A characteristic feature of this family of genes is their rapid induction in response to CK [12].

**Fig. 2.**
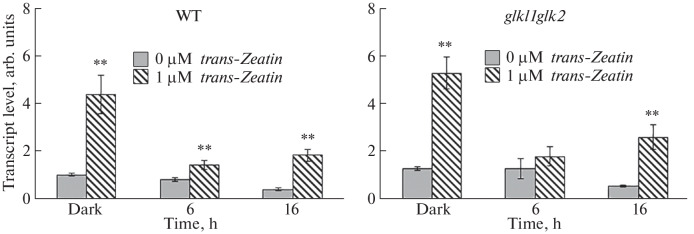
Influence of cytokinin on the content of *ARR5* gene transcripts in wild-type and *glk1glk2* knockout mutant *A. thaliana* seedlings in the dark and during deetiolation. **Significant differences between the mean values of expression in seedlings grown on a nutrient medium without cytokinin vs. expression in plants treated with *trans*-zeatin (*p* ≤ 0.01).

The results of real-time PCR showed an increase in the level of *ARR5* gene transcripts in the wild-type and *glk1glk2* knockout mutant seedlings both in the dark and during cytokinin-dependent deetiolation (Fig. 2). It should be noted that the dynamics of the content of transcripts of the studied gene in the seedlings of the wild type and the *glk1glk2* mutant was similar.

These results led us to conclude that mutations for the GLKs *trans*-factor genes do not affect the perception and, possibly, transduction of the cytokinin signal and that the *glk1glk2* mutant, similarly to the wild-type plants, is sensitive to the exogenous hormone.

**Cytokinin regulates the expression of** ***trans*-factor genes** ***GLK1*** **and** ***GLK2*** **during deetiolation.** Previously, Kobayashi et al. [10] demonstrated cytokinin-dependent induction of the *GLK2* gene in experiments on the deetiolation of *A. thaliana* roots. The selection of only the *GLK2* gene expression for analysis was determined by the tissue-specific expression of the genes of the *GLKs* family: *GLK1* is expressed in only photosynthetic tissues, whereas the level of *GLK1* transcription in roots is below the detection level, and *GLK2* is expressed in both green and non-photosynthetic tissues (roots) [3]. However, it remains unknown whether CK induces the expression of the *GLK1* gene and whether the cytokinin-dependent regulation of the *GLK2* gene is retained during deetiolation of *A. thaliana* seedlings. Based on the data that both genes of the GLKs family are expressed in cotyledon leaves [3], we assumed that GLKs may be involved in the cytokinin-dependent regulatory cascade of gene expression during deetiolation.

**Fig. 3.**
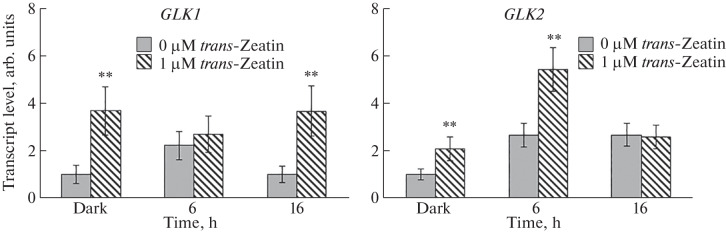
Light and cytokinin regulation of the content of *GLK1* and *GLK2* gene transcripts in 4-day-old *A. thaliana* wild-type seedlings during deetiolation. **Significant differences between the mean values of expression in seedlings grown on a nutrient medium without cytokinin vs. expression in plants treated with *trans*-zeatin (*p* ≤ 0.01).

The analysis of the content of *GLK1* and *GLK2* gene transcripts in the wild-type *A. thaliana* seedlings showed a light-dependent accumulation of the templates of the studied genes (Fig. 3). This induction of the content of *GLK* gene transcripts is consistent with the data of Fitter et al. [3].

Under the light exposure, CK increased the content of *GLK1* and *GLK2* gene transcripts both in the dark and after 6 h (*GLK2*) or 16 h (*GLK1*) of deetiolation (Fig. 3). This result suggests that the *trans*-factors GLK1 and GLK2 may be involved in the cytokinin-dependent regulation of gene expression during deetiolation of *A. thaliana* seedlings.

***trans*-Factors GLKs regulate the cytokinin-dependent expression of the** ***SCA3*** **gene encoding plastid RNA polymerase RPOTp.** To determine whether GLKs family proteins are involved in the regulation of the expression of the *SCA3* gene and RPOTp-dependent plastid genes, the *glk1glk2* double mutant was used.

The cytokinin-dependent activation of expression of GLKs *trans*-factor genes allowed us to assume that these transcription factors are involved in the CK-dependent activation of expression of the RNA polymerase gene *SCA3*. Previously, we demonstrated the regulation of the level of *SCA3* gene transcripts by the cytokinin during *A. thaliana* deetiolation [7]. However, the components of the molecular cascade underlying this positive effect are still unknown.

**Fig. 4.**
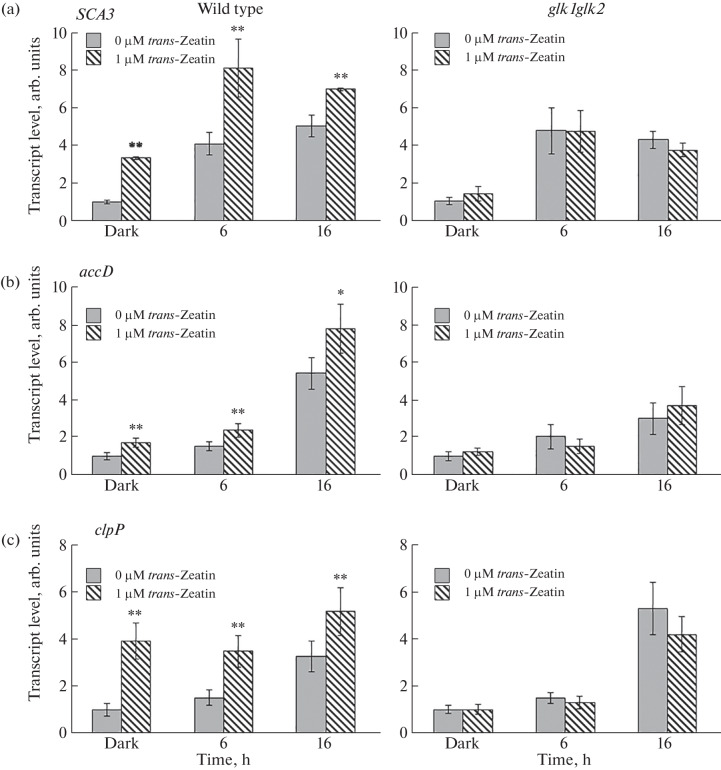
Influence of light and cytokinin on the transcript level of the *SCA3* nuclear gene encoding chloroplast RNA polymerase RPOTp (a) and RPOTp-dependent plastid genes *accD* (b) and *clpP* (c) in 4-day-old wild-type and *glk1glk2* knockout mutant *A. thaliana* seedlings in the dark and during deetiolation. *Significant differences between the mean values of expression in the seedlings grown on a nutrient medium without cytokinin vs. expression in the plants treated with *trans*-zeatin at *p ≤ 0.05 and **p ≤ 0.01.

The results showed (Fig. 4a) that the illumination of etiolated plants stimulated an increase in the level of *SCA3* gene transcripts in the seedlings of both the wild-type plants and the *glk1glk2* knockout mutant. The absence of *trans*-factors GLKs changed neither the profile nor the level of transcripts in the *glk1glk2* plants (Fig. 4a). In turn, in contrast to the wild-type seedlings, the treatment of the *glk1glk2* seedlings with *trans*-zeatin did not lead to an increase in the content of *SCA3* gene templates. The absence of a response to CK indicates the possible involvement of transcription factors GLK1 and/or GLK2 in the realization of the positive effect of CK on chloroplast formation during deetiolation through the activation of expression of the RNA polymerase gene *SCA3*.

***trans*-Factors GLKs mediate the activation of expression of RPOTr-dependent plastid genes** ***accD*** **and** ***clpP*** **by cytokinin during deetiolation of** ***A. thaliana*.** An additional confirmation of the involvement of *trans*-factors GLKs in the cytokinin-dependent activation of the *SCA3* gene expression was the results of RT-PCR regarding the level of transcripts of RPOTp-dependent genes (namely, *accD* and *clpP*) during the cytokinin-dependent deetiolation of the *glk1glk2* mutant. Both genes belong to the group of housekeeping genes: *accD* encodes the β-subunit of acetyl-CoA carboxylase, which is involved in the synthesis of fatty acids, and *clpP* encodes the catalytic subunit of the Clp protease. Since the *accD* gene has only the NEP promoter, the transcription of this gene is performed by the NEP polymerase. The *clpP* promoter contains both NEP and PEP elements, which allows this gene to be transcribed by both NEP and PEP polymerases. However, there are data on a greater contribution of the NEP polymerase to the *clpP* gene transcription [2].

The analysis showed that the illumination of both the wild-type and knockout mutant seedlings leads to an increase in the content of *accD* and *clpP* gene transcripts (Figs. 4b, 4c). Significant differences between the wild-type and *glk1glk2* plants were observed in the dynamics of the mRNA level of the *accD* and *clpP* genes in response to CK during deetiolation: in the maternal line seedlings, exogenous CK increased the content of transcripts of the studied genes at all time points of the experiment, whereas the inactivation of the genes encoding the transcription factors GLKs in the *glk1glk2* mutant led to the absence of cytokinin-dependent regulation (Figs. 4b, 4c).

The absence of a positive effect of CK on the content of transcripts of the *SCA3* gene and two RPOTp-dependent genes *accD* and *clpP* in the *glk1glk2* mutant confirms the involvement of the *trans*-factors GLK1 and/or GLK2 in the realization of the positive effect of CK on the chloroplast formation by controlling the plastid nuclear-coding RNA polymerase.

It is known that CKs have a wide spectrum of functional activity. The initial cytokine signal transduction pathway includes the cytoplasmic AHKs receptors (Arabidopsis Histidine Kinase), AHPs transmitters (Arabidopsis Histidine phosphotransfer Proteins), and 11 ARR (Arabidopsis Response Regulator) type B *trans*-factors [13]. Despite the fact that type B *trans*-factors have 4000 to 8000 direct binding sites for nuclear gene promoters [14], numerous transcriptomic studies indicate a much larger cluster of cytokinin-regulated genes, which suggests the involvement of *trans*-factors of a higher order in the hormone-dependent expression.

The latter include three families of GATA *trans*-factors of nuclear localization: GNC (GATA Nitrate-inducible Carbon-metabolism-involved), GNL/CGA1 (GNC-Like/Cytokinin-responsive GATA factor 1), and GLKs (Golden two-Like) [2, 15]. These regulatory proteins mediate the action of CK on chloroplasts; moreover, the expression of their coding genes is positively regulated by CK [3, 16]. In addition to being involved in the CK-dependent regulation of nuclear gene expression, GNCs and GLKs are involved in the control of biogenesis and division of chloroplast; however, the molecular mechanism of this effect is different. The GNC factor suppresses the transcription of the genes for negative photomorphogenesis regulators *PIFs*, as well as the genes encoding biosynthesis enzymes and *trans*-factors brassinosteroids, thereby initiating photomorphogenesis. Conversely, GLKs activate the transcription of the genes encoding proteins of light-harvesting complexes and enzymes involved in chlorophyll biosynthesis. Our results for the first time showed the involvement of transcription factors GLKs in the cytokinin-dependent activation of *SCA3* gene expression, which significantly deepens the understanding of the mechanisms of regulation of chloroplast biogenesis by the GLKs *trans*-factors.

Another light- and cytokinin-dependent regulator of chloroplast biogenesis is the HY5 *trans*-factor. In the studies on the deetiolation of *A. thaliana* roots, Kobayashi et al. [10] showed the interdependence of the effects of HY5 and GLKs factors. This is confirmed by the fact that the expression of both genes is suppressed by auxin and activated by CK. Overexpression of the *GLK1* and *GLK2* genes leads to an increase in the level of the HY5 protein and the light harvesting complex protein LHCP. Crossing the *hy5-215* mutant with a plant overexpressing *GLK1*_*ox*_ or *GLK2*_*ox*_ compensates to some extent, but does not completely restores, the pale green *hy5-215* phenotype, suggesting that the functionally active HY5 is required for the functioning of GLKs *trans*-factors. In addition, we have previously shown [17] that the cytokinin-dependent expression of the *SCA3* gene during deetiolation is mediated by the HY5 *trans*-factor, and in this study we showed the dependence of the *RPOTp* gene expression on GLKs factors. Apparently, GLKs and HY5 proteins are elements of the same transcriptional cascade that ensures the regulation of the expression of the *SCA3* gene encoding plastid RNA polymerase RPOTp during the cytokinin-dependent deetiolation.
